# A review on the applications of Traditional Chinese medicine polysaccharides in drug delivery systems

**DOI:** 10.1186/s13020-021-00567-3

**Published:** 2022-01-15

**Authors:** Bei Wang, Xianfeng Wang, Zhiwei Xiong, Guanzheng Lu, Weikun Ma, Qinglin Lv, Long Wang, Xiaobin Jia, Liang Feng

**Affiliations:** State Key Laboratory of Natural Medicines, School of Traditional Chinese Pharmacy, China Pharmaceutical University, Nanjing, 211198 People’s Republic of China

**Keywords:** Polysaccharide, Traditional Chinese medicine, Drug delivery systems, Synergistic effect

## Abstract

Traditional Chinese medicine polysaccharides (TCMPs) are plentiful and renewable resources with properties such as biocompatibility, hydrophilicity, biodegradability, and low cytotoxicity. Because the polysaccharide molecular chain contains a variety of active groups, different polysaccharide derivatives can be easily produced through chemical modification. They have been increasingly used in drug delivery systems (DDS). However, the potential of polysaccharides is usually ignored due to their structural complexity, poor stability or ambiguity of mechanisms of actions. This review summarized the applications of TCMPs in DDS around four main aspects. The general characteristics of TCMPs as drug delivery carriers, as well as the relationships between structure and function of them were summarized. Meanwhile, the direction of preparing multifunctional drug delivery materials with synergistic effect by using TCMPs was discussed. This review aims to become a reference for further research of TCMPs and their derivatives, especially applications of them as carriers in pharmaceutical preparation industry.

## Background

There are many limitations of bioactive small molecules for their applicability to human, due to inadequate transportation efficiency and low oral bioavailability [1]. Low bioavailability of some drugs results from their low solubility, instability in gastrointestinal tract (GIT) conditions, inadequate gastric residence time, and difficulty to permeate through the cells through the lipid-bilayer cell membranes of the gut [2, 3]. Recently, DDS plays an important role in increasing the amount of drug loading, controlling the release and improving the bioavailability of the drug [4]. DDS is defined as a technical system in which the distribution of drugs in body can be regulated from the aspects of space, time and dosage. And it aims to achieve the optimum effects of drug molecules in the body, through precise control of their movements. The effective construction of DDS largely depends on the excellent material properties [5]. However, some deficiencies must be considered which are against the design and application of DDS, such as the potential toxicity of carrier materials, the complex preparation of nano-medicine, and the slow response to stimuli. Discovering new and higher-quality carrier materials is always in urgency.

At present, TCMPs attract people’s attention because of their bioactivities such as anti-tumor [6], immunologic enhancement [7] and intestinal microenvironment regulation [8]. For example, *Astragalus* polysaccharide injection, *Ginseng* polysaccharide injection and *Poria cocos* polysaccharide oral liquid have been widely applied in clinic. Furthermore, it is a carrier material with potential due to the inherent properties including nontoxicity, biocompatibility and biodegradability [9–11]. TCMPs are easy to be modified for their huge specific surface area and presence of abundant active groups. TCMPs have the ability to generate a variety of targeted formulations through grafting multiple functional groups, which can improve administration efficiency while lowering adverse effects [12]. Some TCMPs with high affinity for mucosal can prolong the duration period of the drug [13, 14]. TCMPs can also act as absorption enhancers by affecting microbial metabolism or both paracellular and transcellular pathways to promote drug bioavailability [15]. What’s more, unlike chemically inert polysaccharides, the TCMPs have additional biological activities when used in DDS. The TCMPs can be prepared into medicinal excipients through structural modification and molecular optimization. Then it can be used not only as functional component to increase the targeting and bioavailability of the drug, but also as an effective ingredient to increase the curative effect [16, 17]. If effects of synergy and drug-assisted integration can be realized, TCMPs will be used in pharmaceutical preparations in depth.

**Fig. 1:**
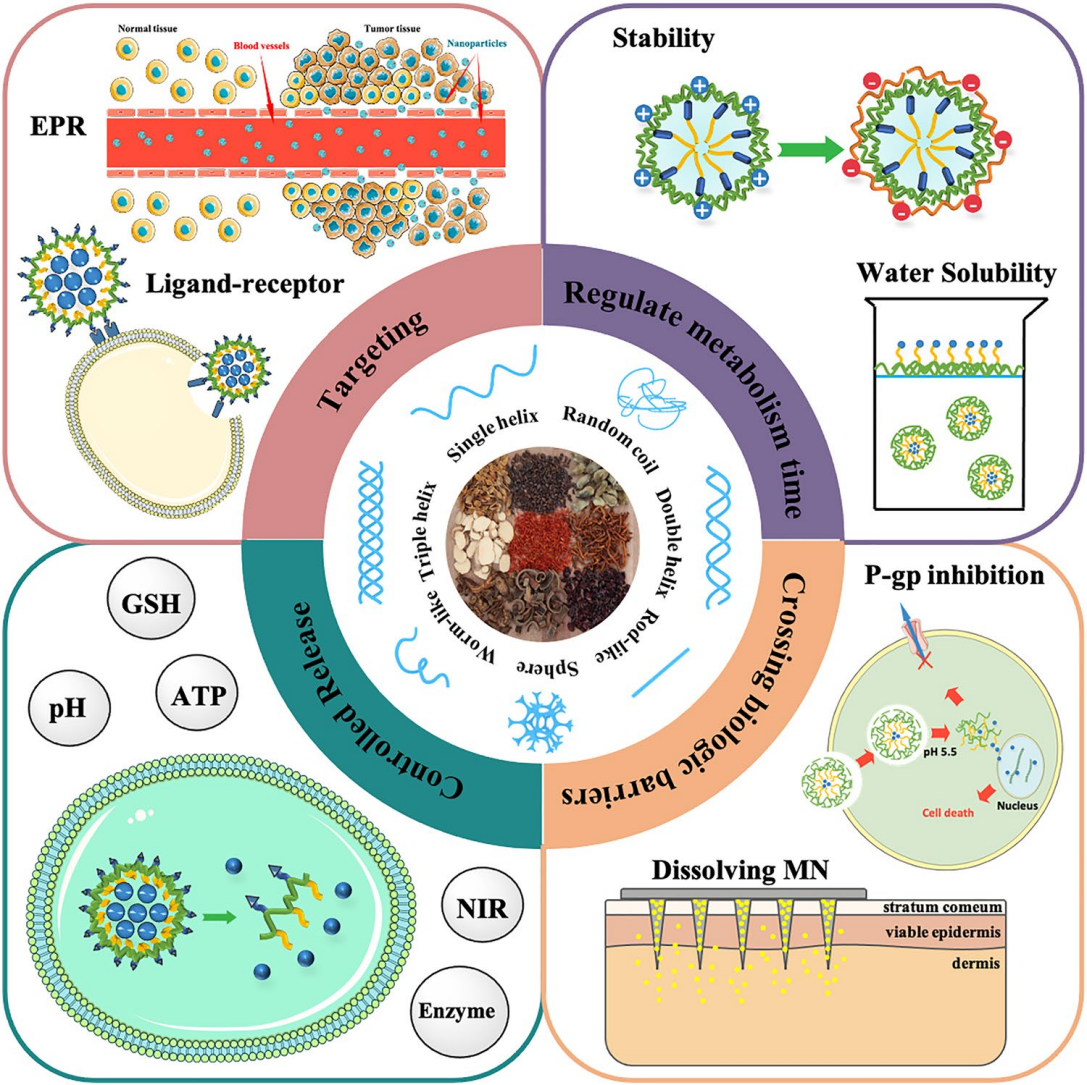
The applications of TCMPs in DDS

However, the polysaccharides derived from Traditional Chinese medicine (TCM) are mostly heteropolysaccharides which consist of different kinds of monosaccharides. Compared with natural polysaccharides such as hyaluronic acid, cyclodextrin and *β*-glucans with triple-helix conformation, it is more complicated to reveal the mechanism, or to develop drugs from TCMPs because of the structural complexity and instability of them. Few reports have summarized the applications of TCMPs in DDS or the relationship between the structure and function of polysaccharides, although there are many works focused on natural polysaccharides as carriers [18]. Most reports about TCMPs are related to pharmacological activities, but few are about the functions of preparations. Therefore, based on the link between polysaccharides structure and function, the general characteristics of TCMPs as carriers were summarized, which may accelerate the development and utilization of TCMPs (see Fig. 1 and Table 1).

**Table 1:** Applications of TCMPs in DDS

Polysaccharide	DDS composition	Form	Function	Application	Ref.
BSP	DTX-SA-BSP	Copolymer micelle	Sustained release, pH sensitive, promoting absorption, enhancing stability	Anticancer	[31]
BSP	SM-hm-BSP	Copolymer micelle	Sustained release	Hepatic-targeted drug delivery	[32]
APS	APS-CMC-BSA	Hydrogel	Sustained release	–	[40]
BSP	PNS-BSP/alginate	Bioadhesive microsphere	Sustained release, synergistic effect	Gastric ulcers	[11, 17]
GLP	RCGDDH NPs	Conjugate	Redox and pH dual-responsive, synergistic effect	Anticancer	[16]
LBP	LBP-5ASA-Pt	Conjugate	Passive targeting (EPR)	Anti-lung cancer	[51]
BSP	Dox@FA-BSP-SA/TPGS	Copolymer micelle	FA-mediated active targeting, synergistic effect	Anticancer	[47]
BSP	DTX-BSP-ss-SA	Copolymer micelle	Redox and pH dual-responsive	Anticancer	[59]
ASP	DOX/ASP-DOCA	Copolymer micelle	pH sensitive, ASGPR-mediated active targeting	Hepatic-targeted drug delivery	[56]
ASP	Cur/ACNPs	Copolymer micelle	ASGPR-mediated active targeting	ALD	[45]
APS	quercetin-APS	Conjugate	Solubilization	–	[65]
VBCP	Baicalin/rhein-VBCP	Copolymer micelle	Solubilization	–	[63]
GPs	Ag-GPs	Nanoparticle	Enhancing stability	Antimicrobial biomaterials	[72]
ASP	AS-PBA/GA-CDB@Cur	Nanoparticle	Enhancing stability, pH sensitive	Antitumor	[83]
RP	CP3-DOX	Nanoparticle	Enhancing stability, promoting absorption, pH sensitive	Antitumor	[79]
BSP	LP-OBSP-CS	Hydrogel	Enhancing stability	Wound healing	[81]
OJP	THSG-OJP	Complexes	Enhancing solubility and stability	–	[64]
BSP	RB-BMNs	–	effective transdermal drug delivery	–	[94]
APS	Se-APS	Nanoparticle	Promoting absorption	Micro nutrient	[89]
LP	LP-Se	Nanoparticle	Promoting absorption	Anticancer	[90]

## Sustained/controlled release

**Fig. 2:**
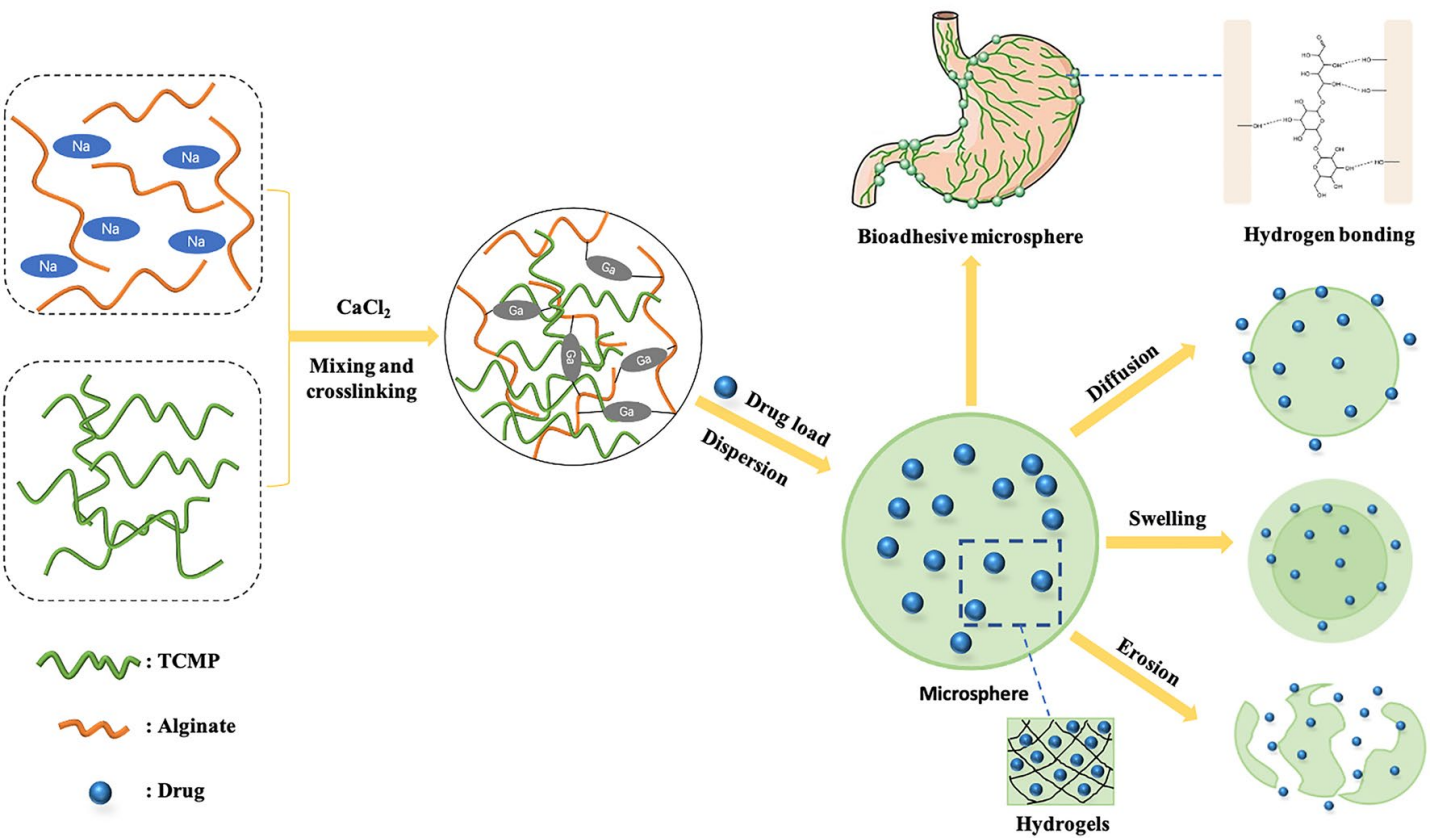
Principle diagram of preparation and drug release for bioadhesive hydrogel microsphere

Sustained/controlled-release preparations can release medicines in a regulated and continuous way, allowing the drug level in the blood or the target area to be maintained in an effective range for a long time. The frequency of administrations can be decreased as a result, and it is appropriate for patients with chronic illnesses who take medications for an extended period of time. TCMPs can be used as carrier materials or polymeric blends to improve the performance of materials because of their features including biodegradability, high-security and biocompatibility. Polysaccharides are composed of various lengths of sugar chains that contain plenty of hydrophilic groups such as hydroxyl, carboxyl, and aldehyde groups, resulting in different structures and characteristics. The chains of TCMPs are easily entangled with glycoprotein, providing the possibility of interaction with other molecules. And then non-covalent bonds such as hydrogen bonds might be formed between the polysaccharide’s active groups and the sugar residues on the glycoprotein oligosaccharide chain. As a result, TCMPs have a strong capacity to adhere to the mucosa. According to the physical properties and chemical structures of polysaccharides, we summarized three types of TCMPs-based DDS with sustained and controlled release effect. Firstly, TCMPs have a high hydrophilicity due to the presence of numerous hydrophilic hydroxyl groups in their structure. Through conjugating some hydrophobic groups onto the molecules of polysaccharides, amphiphilic polymers can be synthesized, which can spontaneously aggregate into micelles in aqueous medium. What’s more, Water-soluble or hydrophilic polymers can form hydrogels through chemical or physical crosslinking. TCMPs have been widely applied in hydrogel materials due to their high hydrophilicity, excellent biocompatibility, and biodegradability. Thirdly, the strong biological adhesion of TCMPs can prolong the drug loading residence duration in the target site [19, 20]. The mechanism of preparation and drug release for bioadhesive hydrogel microsphere is showed in Fig. 2.

### Micelle

Micelles are nanometer-sized aggregates generated by the self-assembly of amphiphilic polymers in aqueous solution. Amphiphilic polymers at low concentrations exist in the form of unimers. Amphiphilic polymer unimers combine to form micelles when the concentration approaches the critical micelle concentration (CMC) [21]. Micelles possess a unique core–shell backbone composed of a hydrophilic shell and a hydrophobic core [22]. Hydrophobic drugs can be encapsulated into the hydrophobic core of copolymer micelles. The creation of functional component delivery systems is made possible by amphiphilic polymers generated following hydrophobic modification of TCMPs. Esterification is a common way for polysaccharides to modulate their amphiphilic properties. The Maillard reaction is another common way to hydrophobically modify polysaccharides [23, 24]. The first phase of the Maillard reaction, a Schiff base reaction, is frequently used to make amphiphilic polymers. For example, *Bletilla striata* polysaccharide (BSP) is mainly composed of (1 → 2)-*α*-D-mannopyranose and (1 → 4)-*β*-D-glucose with the average molecular weight of approximately 100–130 kDa. BSP exhibits many pharmacological activities such as antiulcer, immune-modulatory, wound healing and anti-fibrosis actions [25–29]. A system of stearic acid (SA)-modified BSP micelles was developed for the delivery of docetaxel (DTX) as a model anticancer drug (DTX-SA-BSP). The hydrophobic core of the SA-BSP enveloped lipophilic DTX, and drug release was ascribed to diffusion and dissolution [30]. DTX was gradually released from the DTX-SA-BSP micelles, and a steady release rate was maintained for a relatively long time [31]. Similarly, silymarin (SM) was encapsulated in self-assembled nanoparticles of BSP conjugates modified with SA. The nanoparticles exhibited a sustained-release profile for nearly one week with no obvious initial burst [32].

### Hydrogel

Hydrogels, a polymeric material, have ability to incorporate large amounts of water in their three-dimensional networks [33]. Because of their inherent cellular interaction capability, superior biocompatibility and similarities to extracellular matrices, they have been extensively studied for biomedical applications, including drug delivery [34, 35]. Hydrogels prepared from TCMPs can regulate the strength and hardness of the preparations, thus promoting decomposition and shaping. For example, by crosslinking to varied concentrations of genipin, a variety of hydrogels with different ratios of chitosan (CS) and licorice polysaccharide (LP) were created. The swelling rate rises in the early stages of immersion in buffer as the LP content rises, then falls later. The inclusion of LP lowered the mechanical strength of the hydrogels and slowed their gelation and degradation. Furthermore, when CS is formed as a composite material with water soluble LP, its aqueous solubility improves [36].

Hydrogels can also load enzymes, drugs, antigens, etc. using physical embedding and immobilization techniques. Under the multiple effects of self-diffusion and hydrogel swelling or degradation, the drug is slowly released at the required rate for a long time, thereby greatly improving the utilization rate of the drug [37]. For instance, *Astragalus* polysaccharides (APS) are biological active ingredient with anti-tumor, immunoregulation and hypoglycemic activity [38]. Monosaccharide-composition analysis revealed that APS consisted of glucose, galactose, arabinose, rhamnose, and galacturonic acid with linkages as follows, D-Glcp-(1 → , → 4)-D-Glcp-(1 → , → 2)-L-Rhap-(1 → , D-Araf-(1 → , → 5)-D-Araf-(1 → , → 2,5)-D-Araf-(1 → , → 4)-D-Galp-(1 → [39]. Yan et al. prepared hydrogel with mixing polysaccharide of Snakegourd root/Astragalus and CMC, and used a melt-extruded 3D printer to print the hydrogel into three distinct shaped patches. Pore structure, swelling rate, and degradation characteristics are all favorable in the hydrogels. In addition, the hydrogel has shown to be cell compatible. The findings of the rheology investigation revealed that it had adequate stiffness strength for use in a medication delivery system. The created hydrogels had a sustained release, and different forms of patches had varying drug release rates at the same time, according to the release findings of 3D printed patches with diverse shapes that employed Bovine serum albumin (BSA) as a model drug [40].

Hydrogels prepared from TCMPs are suitable for the delivery of hydrophilic ingredients, and would be synergistic with with the drugs. When BSP and CMC are combined for hydrogel production, a wound dressing with cumulative bioactive effectiveness of both polysaccharides emerges. In this study, increased hydroxyl radical scavenging ability was a strong indicator [41].

### Microsphere

Microspheres are spherical or quasi-spherical particles with a size ranging from 1 to 250 μm made of polymer materials as a carrier. Microspheres can encapsulate different types of drugs, such as small molecules, proteins, and nucleic acids. The medicine can release slowly at a specific location because of the biodegradability and degradation time of the materials, increasing the effective concentration of the drug in the target area [42]. However, there are some drawbacks in the applications of microspheres to oral mucosal administration, such as delayed drug efficacy, instability of drugs in the GIT, and limited affinity for biological tissue. Microspheres incorporated with bio-adhesive TCMPs were expected to improve not only the structural strength of microspheres while maintaining biocompatibility, but also the mucoadhesion of microspheres. These microspheres can be tightly attached to the surface of gastrointestinal mucosa or epithelial cells, increasing the contact between drugs, thereby promoting the absorption of drugs [43]. Muco-adhesive microspheres with alginate and BSP were prepared by ionotropic gelation technique [44] to load *Panax notoginseng* saponin (PNS). The BSP-alginate microspheres are caught in the folds of the stomach due to their tiny particle size, extending the duration PNS spends in the stomach. The addition of BSP to the alginate microsphere improved its flexibility and decreased its rigidity. Furthermore, after blending with BSP, the swelling property, mucin adsorption capacity, and retention rate on the stomach mucosa of alginate matrix improved, enhancing local medication concentration at the lesion site. The prepared PNS-loaded microspheres were round, the release characteristics aligned with the Weibull equation, and the active ingredients were released by diffusion and erosion. The developed microspheres improved the effects of PNS and synergistically exerted the pharmaceutical effects of BSP on acute gastric ulcers [11, 17].

## Targeting

In the last few years, stimulus-responsive nano-drug delivery systems, targeting intracellular and extracellular drug-specific release, have been widely studied. It is expected to improve the specificity and bioavailability of therapeutic drugs, as well as to reduce toxicity and side effects. TCMPs can be utilized as targeted drug carriers directly, or as a carrier material for DDS after modification. TCMPs are easy to undergo a variety of chemical reactions owing to the abundant active groups such as carboxyl groups in the molecular chain. On the one hand, TCMPs can deliver drugs by forming complexes with small-molecule medicines directly. On the other hand, groups with certain functions are grafted onto the molecules of TCM polysaccharide to prepare different types of targeted preparations. Among them, modifying hydrophilic TCMPs with hydrophobic groups (alkyl, aralkyl, and lipid acid) results in an amphipathic substance with improved capacity to capture insoluble anti-cancer medicines [45–47]. The degrees of substitution (DS) values of hydrophobic groups would impact the properties of self-aggregated nanoparticles. For example, the cumulative release percentage of DTX in BSP-SA nanoparticles, the critical aggregation concentration and the average particle sizes all decreased whereas encapsulation efficiency and loading capacity increased along with the DS increase of SA moiety [48]. Meanwhile, there are bonds between the micelle shell and the loaded drug that can be broken in response to certain stimulus [31].

### Passive targeting

**Fig. 3:**
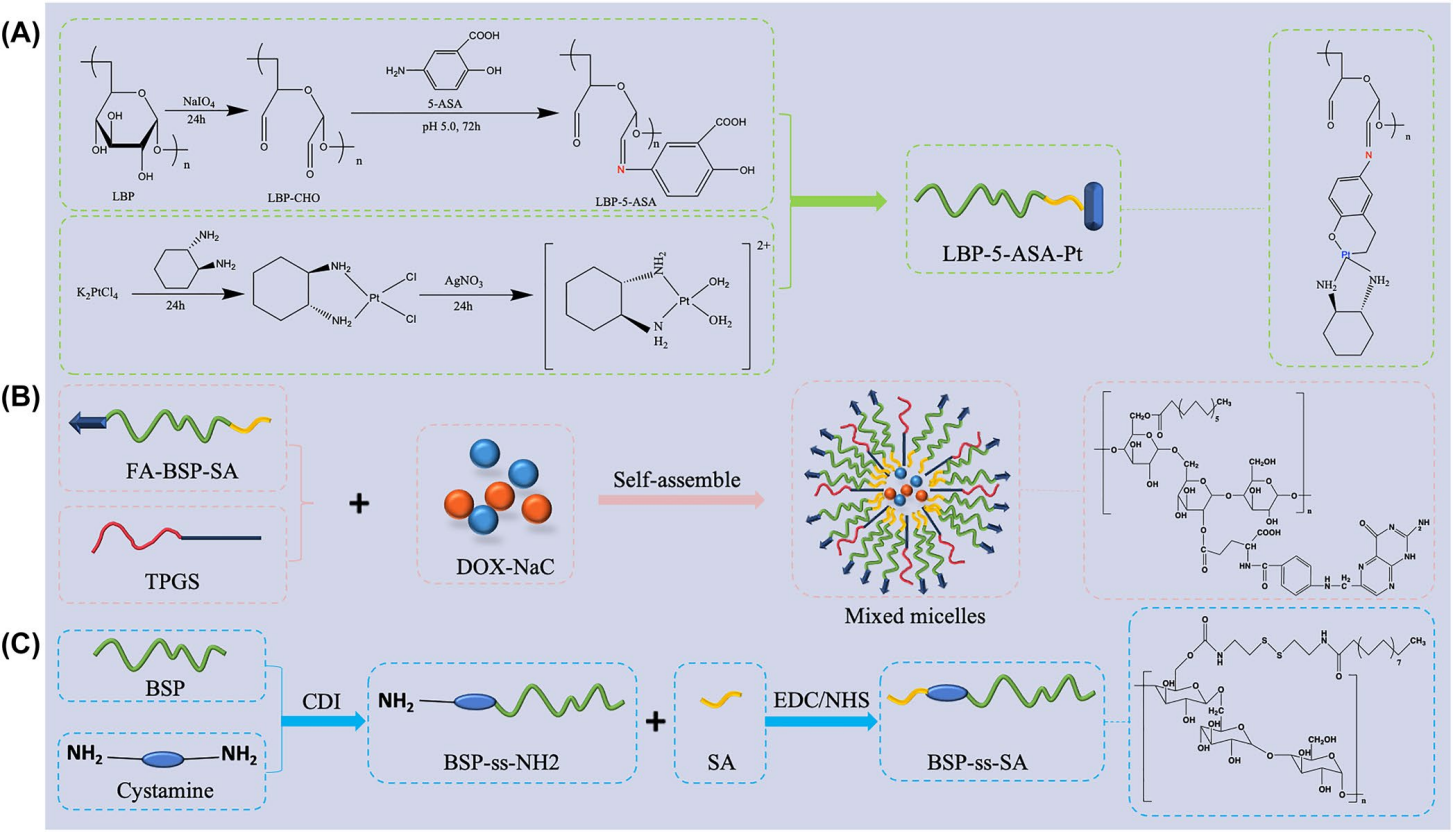
The preparation of TCMPs-based carrier materials of targeted preparations

The distribution of the passive targeting preparation in the body after it is administered intravenously is determined by the size of the microparticles. *Lycium barbarum* polysaccharide (LBP) has been reported to have varieties of biological and pharmacological activities toward hepatitis, diabetes, hyperlipidemia, thrombosis, immunodeficiency, and anti-tumor effects. These glycoconjugates have a high molecular weight ranging from 10 to 2300 kDa. As for structures, literature data provides different results, both in terms of monosaccharide and amino acid residues constituting the glycoconjugates, and relative to glycosidic linkage analysis of glycan backbone, branching sites, and side chains [49, 50]. The aldehyde group of LBP [51] reacted with the amino group of 5-aminosalicylic acid (5-ASA) to form Schiff base. The 5-ASA was used to coordinate Pt complexes in order to build new Pt-polysaccharide drugs conjugates (LBP-5ASA-Pt). The materials fabrication is showned in Fig. 3A. The LBP-5ASA-Pt conjugates could travel to tumor tissue efficiently based on the enhanced permeability and retention (EPR) effect. The Conjugates exhibited certain inhibition specificity to A549 (human lung cancer cell line) and could reduce the damage of normal tissues caused by Pt-based anticancer drugs’ nephrotoxicity and dose-induced toxicity.

### Active targeting

Active targeting preparation uses a modified drug carrier as a “missile” to deliver the drug to the target area. Because the drug-loaded particles are linked to specific ligands or antigens, they can attach to the receptors of target cells after surface modification, altering the normal distribution of particles in the body to reach specific target locations. Therefore, various targeting ligands such as folic acid (FA) [47], growth factors, antibodies, and peptides are introduced into nanoparticles based on TCMPs to improve tumor targeting accuracy. The preparation process of materials is showed in Fig. 3B. It’s worth noting that Angelica *sinensis* polysaccharide (ASP) has a high affinity for asialoglycoprotein receptor (ASGPR), which can accomplish active targeting without adding ligands. The higher galactose content, branching structure, and suitable spatial geometry may contribute to the affinity [52–54]. ASP is water-soluble polysaccharides, and it is mainly composed of glucose (Glc), galactose (Gal), arabinose (Ara), rhamnose (Rha), fucose (Fuc), xylose (Xyl) and galacturonic acid (GalUA). The molecular weight is between 3.2 and 2252 kDa. ASP possesses multiple pharmacological activities, including anti-anaemia, liver protection, antidiabetic activity [55]. ASP [56] was modified with deoxycholic acid to fabricate amphiphilic conjugate (ASP-DOCA), which was synthesized following a one-step method [57]. The blank and drug loaded nanoparticles were prepared by an improved dialysis-sonication method. DOX-loaded nanoparticles (DOX/ASP-DOCA NPs) were absorbed into HepG2 cells via ASGPR-mediated endocytosis, resulting in a stronger anti-proliferation impact than DOX-loaded dextran derivative (DOX/DEX-DOCA NPs), through in vitro cellular uptake. In vivo imaging revealed that DOX/ASP-DOCA NPs preferentially targeted HepG2 tumors via ASGPR, increasing the accumulation of DOX/ASP-DOCA NPs in tumors and producing better anticancer activity compared to free DOX and DOX/DEX-DOCA NPs. Using ASP as a drug carrier material with a liver cancer targeting function can simplify the preparation steps of the carrier. ASP [45] is also used as an instinctive liver-targeting drug delivery carrier in the treatment of acute alcoholic liver damage (ALD). Cholesteryl hemisuccinate (CHEMS) could be directly attached to ASP by an esterification reaction [58]. ASP-CHEMS self-assembled nanoparticles (ACNPs) were prepared by the dialysis-sonication method. Curcumin-loaded ACNPs (Cur/ACNPs) protected the liver from acute ALD by attenuating oxidative stress and were superior to the protective effects of free Cur and the Cur-loaded CHEMS modified-dextran derivative.

### Physical chemistry targeting

**Fig. 4:**
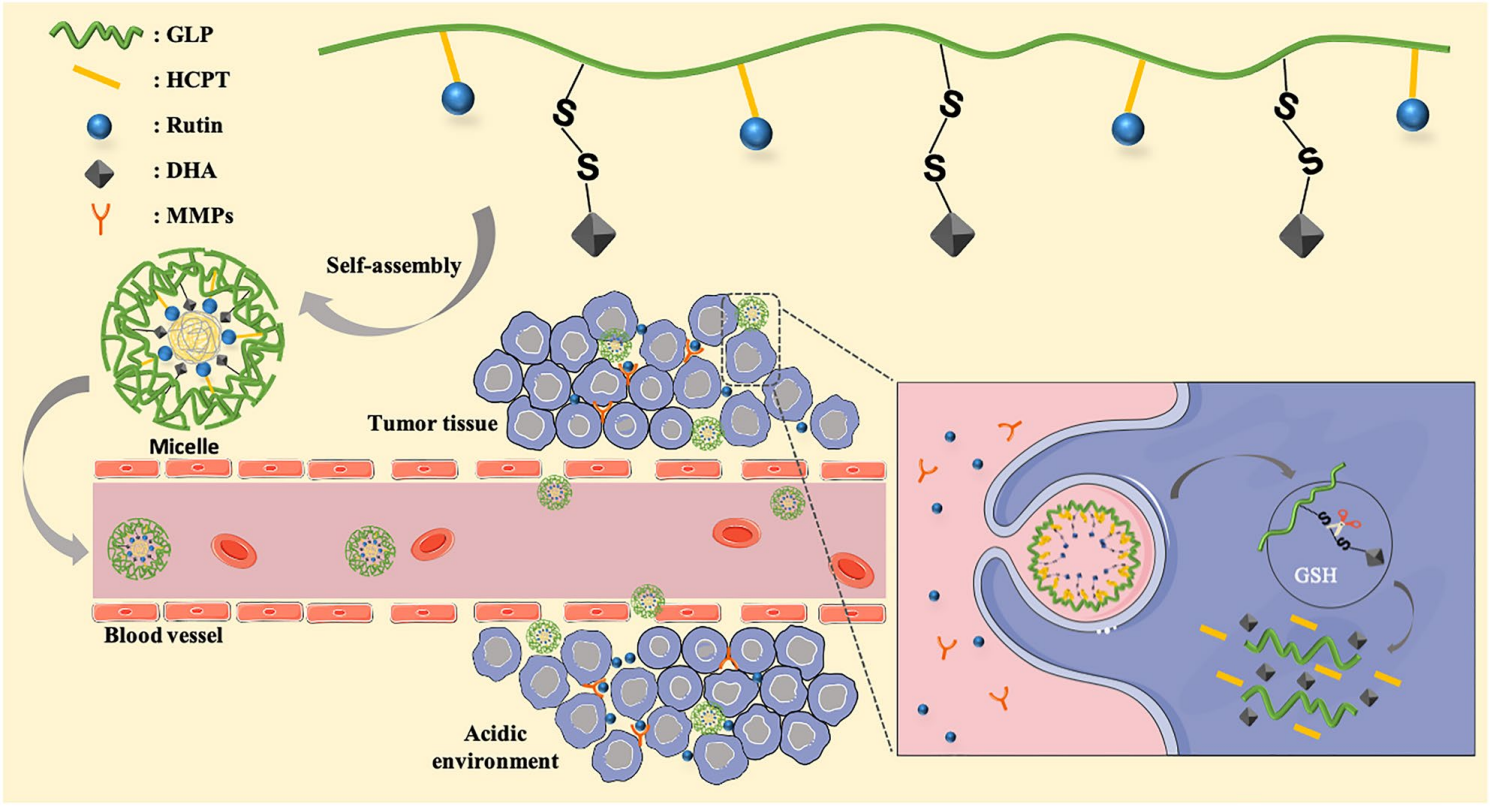
The mechanism of preparation of RCGDDH NPs and schematic diagram of release of anti-cancer drugs in tumor tissue by pH and redox dual-responsive

Physical chemistry targeting preparations could release drugs at specific sites by designing specific carrier materials, which can response to intracellular (pH, redox, ROS) and extracellular (light, heat, magnetic) stimuli. A pH- and redox-dual responsive BSP-based copolymer was synthesized for example [59]. The synthesis of BSP-ss-SA copolymer was mainly divided into two steps, which is showed in Fig. 3C. Firstly, the activated hydroxyl groups of BSP reacted with the amine group of cystamine to synthesize BSP-ss-NH_2_ compound. Secondly, the activated carboxyl group of SA reacted with the amino group of BSP-ss-NH_2_ compound using 1-ethyl-3-(3-dimethyl-aminopropyl) carbodiimide hydrochloride and N-hydroxysuccinimide as catalyzers. BSP-ss-SA micelles were prepared using a dialysis method. DTX-loaded BSP-ss-SA micelles were prepared by emulsification-solvent evaporation method as described in our previous publication [60]. It may be due to the stability of the amide bond and the ester bond in BSP-ss-SA copolymer in pH 7.4 media than the weakly acidic (pH 5.0) environment. The amide bond and the ester bond are easily hydrolyzed under acidic conditions [61]. DTX-loaded BSP-ss-SA micelles showed significant pH-sensitive release behavior. In addition, one characteristic of disulfide bond is its cleavage under the action of reducing agents. BSP-ss-SA micelles exhibited a redox-responsive release property under pH 7.4. The DTX-loaded BSP-ss-SA micelles clearly inhibited the proliferation of HepG2 and 4 T1 cells compared with that of DTX solution. Similarly, *Ganoderma lucidum* polysaccharide (GLP) has been identified as one of the major bioactive components, such as anti-tumour, immune-modulatory, antioxidant, hypoglycaemic. Glucose, mannose, galactose, xylose, fucose and arabinose have been identified in GLP, and only *β*-glucan, a pure glucose polymer, is believed to be one of the active ingredients in GLP [62]. GLP [16] was used as the hydrophilic chain, while the rutin and the dihydroartemisinin (DHA) were connected to GLP as the hydrophobic section by boric ester and disulfide bond, respectively. Besides, 10-hydroxy camptothecin (HCPT) was encapsulated in the nanoparticles. A redox and hydrogen ion concentration pH-dual-responsive DDS (RCGDDH NPs) was developed. The boric ester linkages are first broken and the rutin is released when the RCGDDH NPs arrive at the location of tumor tissue in an acidic environment. Then, using a high concentration of glutathione (GSH), DHA and HCPT are delivered into tumor cells, where the disulfide bonds are rapidly broken, causing nanoparticles to decompose and DHA and HCPT to be released. The mechanism of preparation of RCGDDH NPs and schematic diagram of release of anti-cancer drugs in tumor tissue by pH and redox dual-responsive are showed in Fig. 4. Experiments in vitro and in vivo show that the produced RCGDDH NPs may efficiently kill tumor cells, limit tumor development, and have few side effects. Thanks to the multi-sensitive nano drug delivery technology, new anti-tumor treatment options are now accessible.

## Enhancement of water solubility, stability and permeability of the drug

### Solubility

With the increase of Chinese herbal polysaccharides in the water extract, the extraction rate of some active water-insoluble compounds increased correspondingly, indicating that polysaccharides might be responsible for it [63]. Plenty of researches reported that Chinese herbal polysaccharides could significantly enhance the solubilities of small molecule compounds, but the extent of the enhancement varied widely. TCMPs solubilization of small molecular components follow some rules. The enhancement effect was positively correlated with the quantity of polysaccharides [64], and negatively correlated with the aqueous solubility of small molecule drugs [65]. The greater effect was observed on the more hydrophobic small molecular substance. The smaller effect was observed on the more water-soluble of the substance [66]. What’s more, the polysaccharides with larger molecule weight might contain more glycosidic bonds, which were more complicated and had more branched-chain. So the polysaccharides with large molecular weight might have a stronger solubilization because of possessing more binding sites to interact with drugs [67]. The solubilization coefficients of the flavonoid glycosides were generally superior to flavonoid aglycones [65].

As for solubilization mechanism, on the one hand, polysaccharides, as a water-soluble polymer, can form complexes with drugs to increase the water solubility of drugs. A phase solubility study and DSC characterization indicated that APS could form complexes with flavonoids at 1:1 ratio, and a tendency to improved solubilization at higher association constant values was also observed. The solubilization effects on the different flavonoids were quite different with solubilization coefficient values ranging from 68.88 to 1.75 [65]. On the other hand, it is possible to improve the solubility and dissolution of poorly water-soluble medicines by encapsulating them in carriers such as micelles and liposomes. Polysaccharide from vinegar baked Radix Bupleuri (VBCP), has multiple physiological functions, such as antioxidant activity, hepatoprotective effect, antitumor effect, and immunomodulatory activities [68]. Its backbone chain was composed of 1,5-*α*-L-Araf and 1,3,5-*α*-L-Araf, and the branch chains were 1,5-*α*-L-Araf, 1,4-*β*-D-galacturonic acid or 1,2-*α*-L-Rhap which were linked to the 1,3,5-linked-*α*-L-Araf backbone at C-3 position. VBCP self-assembled to form micelle-like aggregates in water, which can encapsulate water-insoluble constituents through the interaction of both hydrogen bonding and hydrophobic forces [63]. Thermodynamic studies showed that van der Waals forces and hydrogen bonds played major roles between the interaction of drug and polysaccharides [67]. The solubility of Icariin and Baohuoside I can be considerably increased by *Epimedium* polysaccharide, according to our group’s findings. And the mechanism may be related to the formation of micellar complexes between *Epimedium* polysaccharide and insoluble flavonoids.

### Stability

For the oral administration route, the macromolecular nanoparticles must firstly withstand the small pH and digestive enzyme environment in the GIT, and after that they can reach the drug absorption site in the small intestine. Otherwise, the nanoparticle-based oral delivery will be unachievable. Therefore, Efforts should be made to improve the stability of nanocarriers [69]. TCMPs can increase the stability of drugs or carrier materials by forming a complex with the drug or physically embedding the drug. The size of TCMPs-based nanoparticles should kept be less than 200 nm to better avoid rapid clearance by reticuloendothelial system (RES). Besides, the negative charge and excellent solubility of TCMPs make it unfavorable to be attacked by macrophage. In general, the larger the absolute value of a polysaccharide’s potential is, the more stable it is, and uronic acid is mostly responsible for the polysaccharide’s negative charge [70].

Previous researchers have found that some TCMPs can form more stable complexes with small molecule medicines. For example, *Glycyrrhiza* polysaccharides (GPs) exhibit excellent physiological activities, including anti-tumor, immunoregulation, reticuloendothelial system-potentiating, and antioxidation properties [71]. Water-extracted GPs are acidic polysaccharides, and bear some carboxyl groups with a backbone chain composed of *β*-1,3-linked D-galactose residues. GPs [72], which function as a reducing agent and a stabilizer to prevent metal nanoparticles from agglomerating, have a dual impact on the production and stability of Ag nanoparticles. The design of novel antimicrobial biomaterials might be inspired by GPs. What’s more, the grafting of the polysaccharide has been proved to be an effective method to enhance the protein solubility and stability [73–75]. Nanoparticles fabricated by Maillard conjugates were reported that exhibit superior stability to high temperature, pH and ionic strength, which was attributed to the strong steric repulsion of polysaccharide [76, 77]. Radix pseudostellariae (RP) has multiple effects, such as improve immunity and appetite, nourishing vitality and moistening lung [78]. The homologous protein and polysaccharide from RP were used to prepared conjugate with certain glycated degree through dry heating and easily self-assembled into polysaccharide-stabilized protein nanoparticles (CP3) via thermal treatment [79]. The CP3-DOX nanoparticles have great potential in facilitating the efficacy of DOX in cancer cells.

Various studies indicated that hydrogels with stable network structure can encapsulate bioactive ingredients, improving the stability of these components [80]. Plenty of hydroxyl groups of the BSP are prone to oxidation by periodate which could cause the proportion of the aldehyde groups of BSP to increase. Then, the hydrogel material was obtained from the Schiff-base reaction to the aldehyde of OBSP and the amino of CS [81]. This oxidation crosslinking method can enhance the mechanical properties of hydrogel and avoid the potential toxicity of chemical crosslinker or initiators [82]. The *Lactobacillus plantarum* -bound OBSP-CS composite hydrogel (LP-OBSP-CS) as a delivery system can put up a physical barrier to against environmental hazards and improve the probiotics viability.

Anionic TCMPs can be connected with cationic natural polysaccharides through electrostatic attraction or form covalent bonds by chemical reactions. Therefore, the TCMPs can be used as a protective layer to coat the surface of the cationic natural polysaccharides to prevent it from being removed by RES. Subsequently, the drug-carrying system’s stability can be increased. For example, Cur was loaded into the core of a positively charged chitosan oligosaccharide (COS) derivative with mitochondrial targeting ability. The cationic COS nanocarriers’ serum stability is poor, and they are easily eliminated by the reticuloendothelial system. The negatively charged shell based on ASP derivative was wrapped in the surface of the core to protect the drug from the phagocyte elimination in body and the attack of various enzymes. In vitro experiments showed that mitochondrial-targeted core–shell nanoparticles achieved charge-reversal and release more Cur in the acidic tumor microenvironment. After entering into the tumor cells, the lysosomes escaped successfully, and more Cur was transmitted to the mitochondria. The results of in vivo experiments showed that the core–shell nanoparticles efficiently delivered the drug to the tumor site and significantly prolonged the retention time of the drug in the tumor tissue [83].

### Permeability

The bioavailability of a medication is determined not only by its water solubility and stability, but also by the permeability of the gastrointestinal membrane [84]. *Ophiopogon japonicus* polysaccharide (OJP) has many functions such as anti-myocardial ischemia, decreasing blood sugar, boosting immune activity, anti-anaphylaxis. OJP consists mainly of *β*‑fructose and a small amount of *α*‑glucose with the backbones formed by Fruf-(2 → , → 2)-Fruf-(6 → , → 6)-Glcp-(1 → , and → 1,2)-Fruf-(6 → [85]. A study investigated the influences of OJP [64] on 2,3,5,4′-Tetrahydroxy-stilbene-2-*O*-*β*-d-glucoside (THSG). Results showed that OJPs notably enhanced aqueous solubility, and stability of THSG, but slightly decreased the permeability of THSG. In addition, T_max_, C_max_, and AUC(0-t_n_) of THSG were 3.5 fold, 1.45 fold and 2.32 fold higher for THSG-OJP. Thus, OJPs could potentially be used to improve the biopharmaceutical properties and prolong the pharmacological effects of THSG. This finding could provide a reference point for further applications of polysaccharides from herbal medicines. In our group’s study, a homogeneous polysaccharide (RGP2-1) from Red Ginseng (RG), was extracted and we explored its effects on biopharmaceutical properties and solubilization mechanism of baicalin (BA) and glycyrrhizic acid (GA). As result, RGP2-1 with branch structure could significantly improve the solubility and stability of BA and GA in this study, instead of their permeability. What’s more, pharmacokinetics studies showed that RGP2-1 could significantly improve the values of T_max_, C_max_, AUC (0-t) of BA and GA, thus improving the bioavaolability.

## Promoting drug absorption and crossing biologic barriers

Lots of TCMPs have been proven having ability to promote the absorption of small molecule drugs. For example, the transepithelial electrical resistance of excised rat intestinal tissue was considerably decreased by A. *vera* gel polysaccharides, which also improved the transportation of atenolol, a small molecular-blocker, across this tissue [86]. An in vitro study showed that A. vera gel and whole leaf materials were able to reduce transepithelial electrical resistance (TEER) of Caco-2 cell monolayers significantly and also able to enhance the transportation of insulin across this cell culture model. In orally administered TCMs decoctions, microbial metabolism and intestinal absorption are two crucial processes for the absorption and exposure of numerous small molecular components, particularly glycosides. *Ginseng* polysaccharides (GP) were shown to increase Caco-2 cell proliferation and facilitate Rb1 transportation in both directions across the Caco-2 monolayer. Furthermore, the presence of GP accelerated Rb1 microbial metabolism. GP increased Rb1 systemic exposure by increasing microbial deglycosylation and Rb1 absorption via the gut epithelial layer [15]. The Aloe polysaccharide reduced efflux of cimetidine, a substrate of P-glycoprotein [87]. In addition, APS down-regulated the expression of P-glycoprotein in H22 tumor-bearing mice [88], suggesting impact of these botanical polysaccharides on P-glycoprotein-mediated transcellular efflux. These findings pointed out TCMPs' potential as absorption enhancers via both paracellular and transcellular routes.

### Absorption enhancers

Because TCMPs have a high specific surface area and a significant number of terminal hydroxyl groups, they are simple to interact with nanomaterials or cell membranes. Polysaccharides from TCM might be used to modify organic selenium compounds. Using a chelating technique of polysaccharides with sodium selenite, a new water-soluble high Se-enriched APS nanoparticles (Se-APS) [89] was created. Organic selenium compounds offer the benefits of rapid absorption, high bioavailability and low toxicity compared with inorganic selenium compounds. Meanwhile, some researchers employed *Laminaria* polysaccharide (LP) [90] as a modifier and stabilizer in a simple redox process to make stable spherical SeNPs. Modifications improved the cell permeability of selenium nanoparticles. This might explain why LPSeNPs have a stronger anticancer effect. What’s more, the CP3-DOX nanoparticles might act as a P-glycoprotein efflux pump inhibitor and be absorbed into HepG2 cells by clathrin-dependent endocytosis. Compared with free DOX, CP3-DOX nanoparticles significantly enhanced DOX internalization, which was 1.56 fold. The findings imply that CP3 might be a viable option for using as a nanocarrier to enhance anticancer drug absorption in cells [79].

### Dissolving microneedles

TCMPs are biocompatible, biodegradable, and film-forming so that may be utilized safely in the human body. It can be used as a material for preparing dissolving microneedles (MNs). Previously, MNs were primarily made of silicon, metal, and glass, and medication loading was restricted [91–93]. It is probably shattered and left as biohazardous sharp waste in the skin. A dissolving MNs made of BSP [94] breached the skin barrier of the stratum corneum painlessly, enhanced skin permeability and thereby improved transdermal drug delivery effect. After being inserted into the skin, the dissolving MNs gradually dissolved in the interstitial fluid, without sharps waste. Besides, during the manufacture of MNs, most natural materials do not require high temperature treatment, preventing harm to the loaded medicines. Compared with natural materials such as hyaluronic acid [95, 96] and carboxymethyl cellulose [97], BSP has been confirmed to be able to promote wound healing and hemostasis, and have anti-inflammatory, antioxidant, antibacterial activities [28]. It’s utilized not just as a drug carrier, but also as a drug itself when combined with other substances [98]. BSP’s pharmacological actions may aid in the healing of micro-trauma induced by MNs penetration.

## Conclusions and perspectives

The complex interactions between various components in TCM, is the basis for its holistic activity. The interactions between small molecular components and biomacromolecules have received far less attention [99]. Polysaccharides, working as one of the active ingredients in TCM, can be used as both disease-related medications and pharmaceutical excipients. We summarized the applications of TCMPs as carrier materials in the DDS in recent years. It could be concluded that TCMPs have significant advantages and development potential when used in DDS, owing to the following characteristics.

**Fig. 5:**
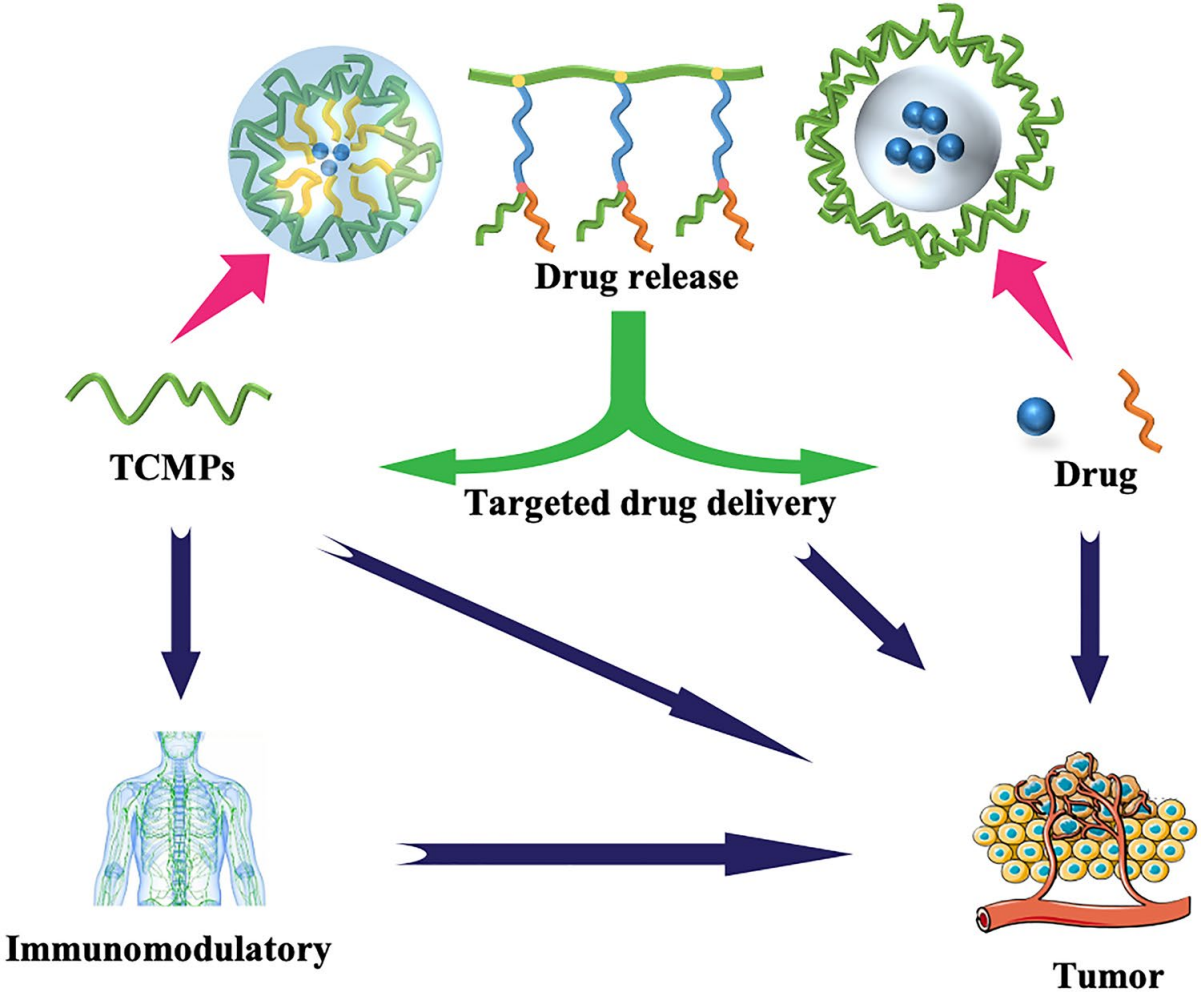
Multifunctional drug delivery materials with synergistic effect by using polysaccharides

TCMPs have biodegradability, safety, and biocompatibility. They can be utilized directly as drug carriers to increase drug delivery efficiency, or in polymer blends to improve the performance of drug-loading material. (1) Because rich hydroxyl groups exist in TCMPs, they have a high degree of hydrophilicity. Firstly, TCMPs, as water-soluble polymers, can form complexes with a medication to increase its water solubility. Secondly, hydrophilic polysaccharides from TCM can form hydrogels through certain chemical or physical crosslinking. Drugs were encapsulated in the TCMPs and diffused into the body fluid through the gel layer to achieve the effect of sustained release. Thirdly, conjugating hydrophobic groups onto the molecules of hydrophilic polysaccharides to synthesize amphiphilic polymers can spontaneously aggregate into micelles in aqueous medium. The hydrophobic core of copolymer micelles can be used to encapsulate hydrophobic medicines, which can improve the water solubility of small molecule drugs. Furthermore, because a hydrophilic shell consisting of macromolecular polysaccharides is coated on the drug surface, it can escape enzyme digestion in the GIT, improving the stability of small molecule medicines. In addition, due to the shell-core structure of micelles, encapsulated drugs can be released gradually to achieve controlled release. What’s more, targeting preparations can be prepared by forming environmentally sensitive release bonds between the shell and core of micelles. (2) TCMPs are active to be modified and then interact with nanomaterials or cell membranes for their huge specific surface area and presence of abundant active groups. Firstly, TCMPs can form complexes with drugs directly or graft various functional groups to create different types of targeting formulations. Secondly, TCMPs chains are readily intertwined with mucosal glycoproteins. The active hydroxyl group of the polysaccharide subsequently creates a hydrogen bond with the sugar residue on the mucosal glycoprotein oligosaccharide chain. As a result, TCMPs have a high affinity for mucosal adhesion, allowing for controlled release. Thirdly, the grafting of the TCM polysaccharide as carrier materials has been proved to be an effective method to enhance the solubility and stability of protein. TCMPs can also be used as stabilizers and absorbent promoters for metal drugs by forming nanoparticles. (3) The negative charge of TCMPs helps it to avoid macrophage attack. Therefore, the anionic TCMPs may be employed as a protective layer to coat the surface of the cationic natural polysaccharides through electrostatic attraction, increasing the stability of the drug-carrying system. (4) When used as a pharmaceutic adjuvant in DDS, TCMPs can sometimes behave as active agents due to their bioactivity, which has a synergistic effect, particularly in the area of anti-tumor. The use of immune cells to destroy tumors is more effective than nanoparticles designed to overcome the barrier of vascular endothelial cells. TCMPs have an anti-tumor impact in two ways. One method is to act directly on tumor cells, causing them to apoptose and so achieving an anti-tumor impact [100]. The other is to boost the body's immunity and encourage it to produce immunological components in order to accomplish anti-cancer effects [101–105]. Therefore, it is predicted that utilizing the immunomodulatory properties of TCMPs and preparing them into carrier materials would have a synergistic impact in the treatment of malignancies. The multifunctional drug delivery materials with synergistic effect by using TCMPs are showed in Fig. 5.

At present, the research on polysaccharides of TCM is still in the initial stage with many deficiencies. TCMPs have poor stability, thus they are prone to structural changes during the extraction, separation and purification process. Moreover, the polysaccharides prepared by different researchers in different batches vary, so it is difficult to reproduce. Due to the difficulty of structural analysis, only the primary structure can be inferred. The advanced structure information is even rarer. Compared with natural polysaccharides, TCMPs are less used in pharmaceutics and clinic, and there is a lack of research on the relationship between structure and function. When TCMPs are employed as carrier materials in DDS, there are still issues with low drug loading and encapsulation rate. It's worth investigating if the newly created complex will affect the drug’s efficacy by altering the drug's and polysaccharide’s original properties. Because the synthesis process is complicated and uses a variety of organic solvents, safety issues need to be paid more attention.

In view of the above problems, the reproducibility of uniform polysaccharide preparation can be ensured by forming a stable and repeatable preparation process. Through the combined use of multiple technologies, interdisciplinary methods are further depended in the structure analysis of TCMPs. Then, according to the relationships between structure and function, the rules can be summarized and more suitable carrier materials can be found for various needs. Synergistic polysaccharide pharmaceutical excipients can be developed under the guidance and enlightenment of TCM theory, especially the unique advantages of TCMPs with biological activities.

## Data Availability

All data included in this article are available from the corresponding author upon request.

## Abbreviations

TCM: Traditional Chinese medicine
TCMPs: Traditional Chinese medicine polysaccharides
DDS: Drug delivery system
GIT: Gastrointestinal tract
CMC: Critical micelle concentration
SA: Stearic acid
BSP: *Bletilla striata* Polysaccharide
DTX: Docetaxel
BSA: Bovine serum albumin
PNS: *Panax notoginseng* Saponin
GLP: *Ganoderma lucidum* Polysaccharide
DHA: Dihydroartemisinin
HCPT: 10-Hydroxy camptothecin
pH: Hydrogen ion concentration
GSH: Glutathione
LBP: *Lycium barbarum* Polysaccharide
5-ASA: 5-Aminosalicylic acid
EPR: Enhanced permeability and retention
FA: Folic acid
ROS: Reactive oxygen species
DS: Degrees of substitution
DOX: Doxorubicin
ASGPR: Asialoglycoprotein receptor
ASP: *Angelica* Polysaccharide
ALD: Alcoholic liver damage
CHEMS: Cholesteryl hemisuccinate
Cur: Curcumin
APS: *Astragalus* Polysaccharide
VBCP: Vinegar baked Radix Bupleuri
GPs: *Glycyrrhiza* Polysaccharides
RES: Reticuloendothelial system
COS: Chitosan oligosaccharide
RP: *Radix pseudostellariae*
OJP: *Ophiopogon japonicus* Polysaccharide
THSG: 2,3,5,4′-Tetrahydroxy-stilbene-2-*O*-*β*-d-glucoside
AUC: Area under the curve
RG: Red Ginseng
BA: Baicalin
GA: Glycyrrhizic acid
MNs: Dissolving microneedles
RB: Rhodamine B
LP: *Laminaria* Polysaccharide
TEER: Transepithelial electrical resistance
GP: *Ginseng* Polysaccharides
Glc: Glucose
Gal: Galactose
Ara: Arabinose
Rha: Rhamnose
Fuc: Fucose
Xyl: Xylose
GalUA: Galacturonic acid
